# Pcf1, a large subunit of CAF-1, required for maintenance of checkpoint kinase Cds1 activity

**DOI:** 10.1186/2193-1801-3-30

**Published:** 2014-01-17

**Authors:** Tatsuki Kunoh, Toshiyuki Habu

**Affiliations:** Laboratory of Molecular Pharmacogenomics, School of Pharmaceutical Sciences, Kinki University, Higashiosaka, 577-8502 Japan; Radiation Biology Center, Kyoto University, Kyoto, 606-8501 Japan

**Keywords:** Chromatin, Pcf/CAF-1, Cds1, Clr6-HDAC, Histone H4, Fission yeast

## Abstract

Highly conserved chromatin assembly factor 1 (CAF-1) is required for histone deposition onto newly synthesized DNA to maintain genome stability. This study shows that the fission yeast Pcf1, the large subunit in CAF-1, is crucial for maintaining checkpoint kinase Cds1. Chromatin recruitment of Cds1 is enhanced by Pcf1 overproduction but is attenuated by the Δ*pcf1* mutation. Mutation of acetylation sites in the histone H4 tail abrogates the chromatin recruitment of Pcf1, which resembles that of Cds1 as reported previously. The present results provide evidence that chromatin recruitment of Pcf1, moderated by Clr6-HDAC activity, is essential for inactivating Cds1.

## Background

In eukaryotes, DNA is assembled into a nucleoprotein complex called chromatin. The fundamental repeating unit of chromatin is the nucleosome in which DNA is wrapped around an octamer of core histone proteins including two H2A/H2B dimers and an (H3/H4)_2_ tetramer (Ridgway and Almouzni 2000). The nucleosome is assembled primarily during DNA replication. Newly synthesized histones are deposited onto duplicated DNA along the replication fork with the aid of specialized assembly proteins (Ehrenhofer-Murray 2004). Nucleosome assembly is also required for DNA repair and transcription. After nucleotide excision repair or double-strand break repair, the nucleosome is reassembled at the site of repaired DNA (Linger and Tyler 2005; Lewis et al. 2005; Nabatiyan et al. 2006). Transcription is generally thought to correlate with a reduction of histone density on the DNA template allowing for the passage of the transcription machinery. It was reported that nucleosomes were selectively removed and replaced by chromatin remodeling factors and histone chaperones (Li et al. 2007; Saunders et al. 2006; Ehrenhofer-Murray 2004).

The conserved CAF-1 (chromatin assembly factor-1) protein complex was first identified in human cells by its ability to stimulate nucleosome assembly *in vitro* (Smith and Stillman 1989). This protein complex, which is associated with a DNA-sliding clump protein PCNA (Proliferating Cell Nuclear Antigen), localizes in the replication fork and deposits a newly synthesized acetylated form of histones H3 and H4 (Shibahara and Stillman 1999). Once assembled in nucleosomes, the histones are promptly deacetylated by histone deacetylases (HDACs). However, both CAF-1 and acetylated-H4 are transiently maintained at the late replication foci, suggesting that CAF-1 and HDACs might interact during chromatin maturation (Taddei et al. 1999). Indeed, CAF-1 plays an essential role in maintaining constitutive heterochromatin in yeast (Huang et al. 2007). Despite the established role of CAF-1 in replication-coupled nucleosome assembly, deletion of any of the three CAF-1 genes has minimal adverse effect on normal growth in yeast (Kaufman et al. 1997), suggesting that other histone chaperones such as Asf1 (anti-silencing factor 1) and HIR/HIRA (histone regulation) may function in H3/H4 assembly cooperatively with CAF-1 (Tamburini et al. 2006; Greenall et al. 2006).

The DNA replication checkpoint has a surveillance function that regulates origin firing, maintains the integrity of the stalled replication fork, and prevents cells from proceeding to mitosis before completion of the DNA replication (McNeely et al. 2013). The replication checkpoint pathway is highly conserved in eukaryotes. In mammalian cells an initial defect is sensed by a protein kinase, termed ATR, which transmits signals to Chk2 effector kinase. In fission yeast, the replication checkpoint requires the ATR ortholog Rad3 and Chk2 ortholog Cds1 (McGowan and Russell 2004). In budding yeast, the checkpoint effector kinase Rad53 directly interacts with Asf1 and regulates chromatin assembly to promote cell survival against DNA damage and replication block (Sharp et al. 2005). Although little is known about the mechanism, CAF-1 is associated with the full activation of the Chk1-dependent checkpoint pathway upon a replication stress in vertebrate cells (Takami et al. 2007). These reports indicate the importance of histone assembly in the S-phase checkpoint response. In budding yeast, hyperacetylation of H3K56, a hallmark of replication-associated lesions, results in activation of Rad53 (Maas et al. 2006). Deacetylation of H4 tail is required for inactivation of Cds1 upon replication stress in fission yeast (Kunoh et al. 2008), suggesting that the acetylation status of histones could affect the checkpoint response. However, how the acetylation status affects histone assembly and thereby checkpoint maintenance in response to the replication block remains unsolved.

In the present paper, we show that Pcf1, the large subunit of fission yeast CAF-1, is required for chromatin organization, maintenance of Cds1 activity, and its chromatin recruitment. Further, chromatin recruitment of Pcf1 depends on the acetylation status of the H4 tail regulated by the Clr6-HDAC, so that it may contribute to the checkpoint inactivation after replication stress.

## Results

### Pcf1, the large subunit of CAF-1, is involved in chromatin organization and interacts genetically with the replication checkpoint pathway component Cds1

**Figure 1:**
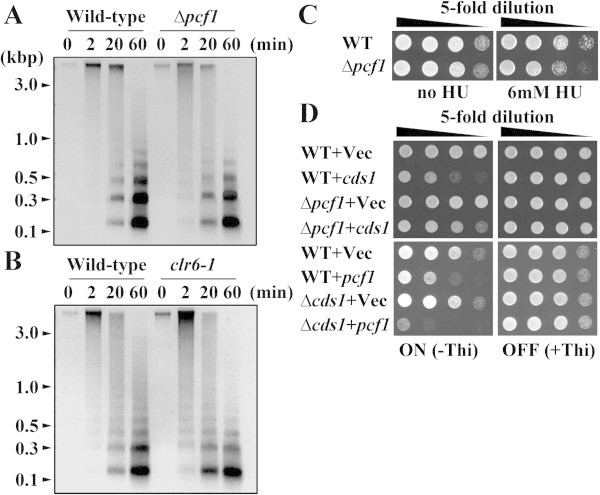
**Electrophoresis patterns of MNase-digested DNA of** Δ***pcf1*** **mutant and wild type cells, their sensitivity to hydroxyurea (HU) and genetic interactions between** ***cds1*** ^**+**^ **and** ***pcf1*** ^**+**^ **. (A, B)** Agarose-gel electrophoresis patterns of DNA prepared from enzyme-digested chromatin of wild type and Δ*pcf1* mutant **(A)** and *clr6-1* mutant **(B)** cells. Note the early appearance of smaller DNA fragments, corresponding to nucleosomes, from digested chromatin of both Δ*pcf1* and *clr6-1* mutants relative to that of the wild type. **(C)** Sensitivity of wild type and Δ*pcf1* mutant cells to HU. Both cells grew similarly on the plates without HU regardless of its dilution, whereas Δ*pcf1* mutant cells grew slowly relative to wild type cells on plates amended with 6 mM HU. **(D)** Growth of wild type, Δ*pcf1* and Δ*cds1* mutant cells harboring the empty vector (Vec), pREP-*cds1* ^+^, or pREP-*pcf1* ^+^ in the presence or absence of thiamine. For a detailed comparison of the growth of these cells, see the text.

During DNA replication, histone deposition is critical for chromatin organization. Among histone chaperones, CAF-1 is considered to be responsible for this process in vertebrate cells (Taddei et al. 1999). In fission yeast cells, proteins homologous to the CAF-1 subunits were shown to form a complex that associates with PCNA (Dohke et al. 2008). Nevertheless, whether CAF-1 is required for chromatin organization in fission yeast remains unclear. To answer this query, we isolated bulk chromatin from wild type and Δ*pcf1*, the mutant lacking *pcf1*^+^ gene encoding the large subunit of CAF-1 (Dohke et al. 2008), then digested the chromatin with micrococcal nuclease (MNase). As illustrated in Figure 1A, nucleosome-corresponding DNA fragments with fewer base pairs appeared earlier from the digested chromatin of Δ*pcf1* mutant than that of the wild type. By 2 min after digestion, DNA fragments had already appeared in Δ*pcf1* mutant but not in the wild type. The intensity of the bands corresponding to the oligo-nucleosomes was stronger in the wild type than in the Δ*pcf1* mutant at 20 and 60 min after digestion. This earlier digestion of bulk chromatin in the Δ*pcf1* mutant was confirmed in repeated experiments. As a positive control, *clr6-1* mutant was subjected to MNase digestion, since it was hypothesized that Clr6-HDAC participated in global deacetylation of histones, affecting chromatin maturation throughout the genome (Grewal 2000). The pattern of small fragment DNAs digested by MNase in *clr6-1* mutant was quite similar to that in the Δ*pcf1* mutant (Figure 1A, B). Thus, the bulk chromatin from Δ*pcf1* and *clr6-1* mutant cells is likely to be more sensitive to MNase relative to that from the wild type cells. The results suggest that the global chromatins may readily relax in the Δ*pcf1* mutant cells, although such a conclusion cannot be drawn until the band intensities of Figure 1A are quantified.

Because deletion of *pcf1*^+^ did not affect normal growth of yeast cells (Figure 1C, left), CAF-1 including Pcf1 might function cooperatively in chromatin assembly with other histone chaperones such as Asf1 and HIR/HIRA proteins as already mentioned. On the other hand, the Δ*pcf1* mutant cells grew slowly relative to the wild type cells under the DNA replication stress imposed by hydroxyurea (HU) (Figure 1C, right), suggesting that Pcf1 might be involved in maintenance of genome stability during the slowed DNA replication in fission yeast.

To determine whether Pcf1 is involved in the DNA replication checkpoint pathway, we examined the genetic interaction of Pcf1 with an effector kinase Cds1. Consistent with a previous report (Boddy et al. 1998), overexpression of *cds1*^+^ led to slow growth of wild type cells, when the *nmt1* promoter was activated under a thiamine-free condition (WT + Vec vs. WT + *cds1* in the upper left of Figure 1D). However, in the thiamine-free condition, cell growth notably improved with deletion of *pcf1*^*+*^ (WT + *cds1* vs. Δ*pcf1* + *cds1*), although it was slower than that of the vector control (Δ*pcf1* + Vec vs. Δ*pcf1* + *cds1*). On the other hand, the slow growth enforced by overexpression of *pcf1*^+^ in wild type cells (WT + Vec vs. WT + *pcf1* in the lower left of Figure 1D) was markedly enhanced by deletion of *cds1*^+^ (WT + *pcf1* vs. Δ*cds1* + *pcf1*). By contrast, in the presence of thiamine, growth of these cells was not affected (Figure 1D, upper and lower right), since *cds1*^+^ or *pcf1*^+^ driven by *nmt1* promoter was expressed at low level. Apparently, a strong genetic correlation exists between *pcf1*^*+*^ and *cds1*^*+*^. These results directed us to examine whether Pcf1 was involved in the DNA replication checkpoint response.

### Pcf1 required for maintenance of Cds1 activity

**Figure 2:**
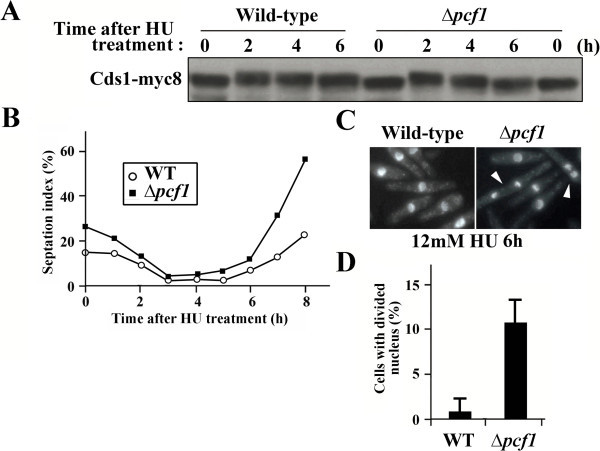
**Disturbance of the activation of the DNA replication checkpoint by deletion of** ***pcf1*** ^**+**^ **. (A)** Mobility of Cds1 prepared from wild type and Δ*pcf1* mutant on SDS-PAGE at various times after HU treatment. **(B)** Time course of septum formation in cells (septation index) in Δ*pcf1* mutant (closed square) and wild type cells (open circle) after HU treatment. **(C)** DAPI-stained cells of wild type and Δ*pcf1* mutant 6 h after HU treatment. Arrowheads indicate cells with a segregated nucleus. **(D)** Incidence of cells with a segregated nucleus 6 h after HU treatment.

Tanaka et al. (2001) reported that the phosphorylated Cds1 migrated slowly on SDS-PAGE when the DNA replication checkpoint was activated. In the present study, therefore, this mobility shift was used as a marker to examine how the lack of Pcf1 affected the DNA replication checkpoint response. The mobility pattern of the cell extracts from the wild type and Δ*pcf1* (Figure 2A) showed that phosphorylation of Cds1 in the former cells was sustained for 2–6 h after the HU treatment, whereas for only 2–4 h in the latter cells. These results obviously show that Pcf1 plays a key role in keeping the active state of Cds1. The septation index (Figure 2B), micrographs (Figure 2C) and count data (Figure 2D) jointly show that the Δ*pcf1* mutant cells proceeded to G2/mitosis earlier than the wild type cells did in the presence of HU, suggesting that Pcf1 might be crucial for checkpoint maintenance upon replication stress.

### Cds1 is recruited to chromatin in a Pcf1-dependent manner

**Figure 3:**
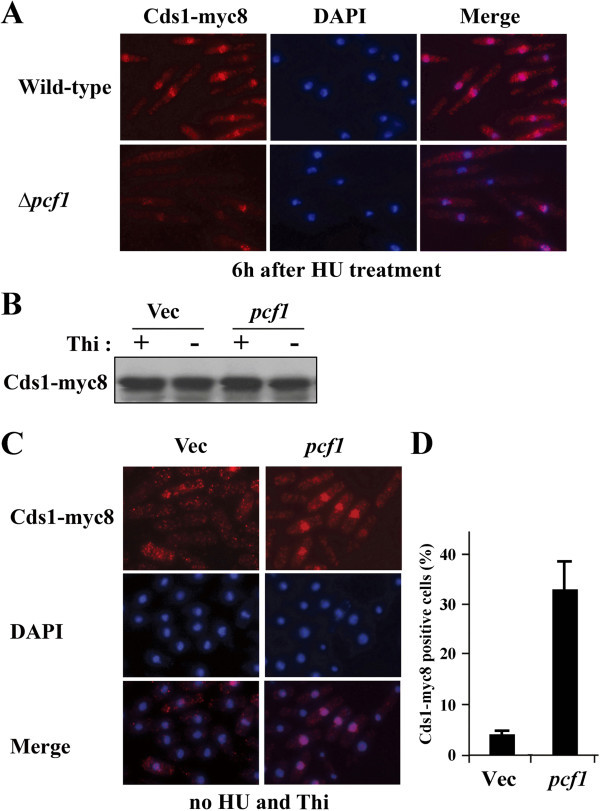
**Pcf1-dependent recruitment of Cds1 to chromatin. (A)** Immunofluorescent images of antibody-labeled Cds1, DAPI-staining images and their merging images of wild type and Δ*pcf1* mutant cells. Cells were harvested 6 h after HU treatment and subjected to Triton extraction, followed by staining with the anti-c-myc antibody and DAPI. **(B)** Mobility of Cds1 from wild type cells harboring the empty vector or pREP-*pcf1* ^*+*^ in the presence or absence of thiamine (Thi). Note that no mobility shift occurred upon overexpression of *pcf1* ^+^. **(C)** Immunofluorescent images of antibody-labeled Cds1 after Triton extraction, DAPI-stained images, and their merged images for wild type harboring the empty vector (Vec) or pREP-*pcf1* ^+^ in the absence of thiamine. **(D)** Incidence of cells with Cds1-myc8-positive nuclei after Triton extraction followed by staining with the anti-c-myc antibody.

Binding stability of Cds1 to chromatin was examined by Triton extraction and immunofluorescence as reported previously (Kunoh et al. 2008). This technique is able to detect the chromatin-bound Cds1 in the cells after the Triton extraction. We previously reported that Cds1 was removed during Triton extraction of asynchronously cultured cells but not from cells in which the replication checkpoint was activated by HU because Cds1 remained bound to chromatin (Kunoh et al. 2008). As expected, immunofluorescence images of the wild type and Δ*pcf1* cells 6 h after HU treatment (Figure 3A) illustrated that Cds1 was tightly bound to DAPI-stained chromatin in the wild-type (chromatin binding affinity of Cds1 = 83%) but not in the Δ*pcf1* mutant (19%), which is consistent with the aforementioned evidence that Cds1 was still activated 6 h after HU treatment in wild type cells but not in Δ*pcf1* mutant cells as shown in Figure 2A. Because these results support the conclusion that Pcf1 is required to maintain Cds1 activity, we assumed that overexpression of *pcf1*^*+*^ could activate Cds1. However, overexpression of *pcf1*^*+*^ did not cause the mobility shift in the absence of thiamine (Figure 3B). Nevertheless, it resulted in tight binding of Cds1 to chromatin even in the absence of HU (Figure 3C and D), indicating that overexpression of *pcf1*^*+*^ does not cause phosphorylation of Cds1 but rather recruitment of Cds1 to chromatin.

Figure three B of Kunoh et al. (2008) of an immunoblot using the tetra-Ac histone H4 antibody illustrated that acetylation of H4 in the wild type was abrogated after HU treatment and that, in the same blot, the band intensity of the histone H4.2 K8A K16G mutant was weak, similar to the wild type after HU treatment, whereas the latter was higher than the former when cells were cultured asynchronously. In contrast, acetylation of H4 was not abrogated in *clr6-1* mutant. These results led us to consider that at least acetylation at K8 and/or K16 residues of H4 tail, removed by Clr6-HDAC, was associated with response to HU treatment. Furthermore, phosphorylation and chromatin recruitment of Cds1 in the *clr6-1* mutant were sustainably maintained once activated. Conversely, the H4.2 K8A K16G mutant failed to keep the checkpoint active due to extraordinary phosphorylation and reduction in the recruitment of Cds1 to chromatin upon replication stress (Kunoh et al. 2008), which resembles the situation in the Δ*pcf1* mutant (Figures 2A and 3A).

### Pcf1 recruitment to chromatin is regulated by the acetylation state of H4

**Figure 4:**
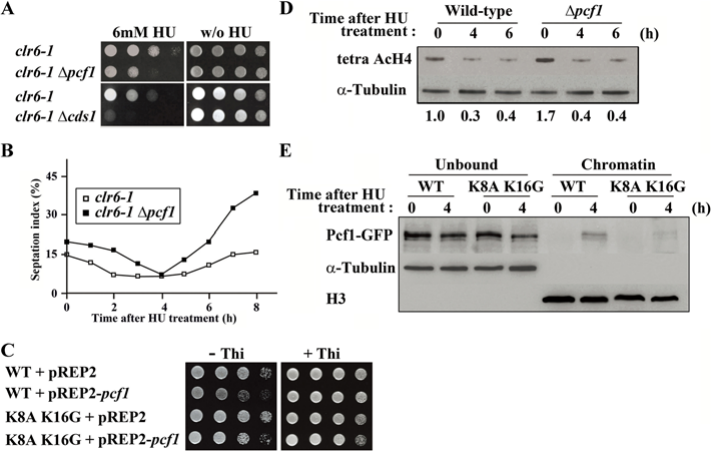
**Recruitment of Pcf1 to chromatin regulated by the acetylation state of H4. (A)** Cell growth of *clr6-1* single mutant and *clr6-1* Δ*pcf1* and *clr6-1* Δ*cds1* double mutant cells in the presence or absence of HU. Note the slower growth of the double mutants relative to that of *clr6-1* single mutant in the presence of HU. **(B)** Septation index of *clr6-1* single (open square) and *clr6-1* Δ*pcf1* double mutant (closed square) at various times after the HU treatment. **(C)** Cell growth of wild type and H4.2 K8A K16G mutant cells harboring the empty vector or pREP1-*pcf1* ^+^ in the presence or absence of thiamine. Note that slow growth by Pcf1 overproduction in wild type cells was suppressed by H4 K8A K16G mutation. **(D)** Immunoblotting of acetylated H4 (tetra AcH4) from the wild-type and Δ*pcf1* mutant cells at various times after the HU treatment. As a loading control, an anti-α-tubulin antibody was used. **(E)** Immunoblotting of chromatin-bound and -unbound fractions prepared from cell extracts of wild-type and H4.2 K8A K16G mutant cells at indicated times after HU treatment.

We next tested whether Clr6-HDAC and Pcf1 functioned to regulate Cds1 in the same pathway. Disruption of *pcf1*^*+*^ made the *clr6-1* mutant cells extremely sensitive to HU, similar to the case in the Δ*cds1 clr6-1*-double mutant (Figure 4A, left). In addition, on the basis of the septation index (Figure 4B), the deletion of *pcf1*^+^ directed the *clr6-1* mutant cells toward G2/mitosis upon replication stress, similar to the deletion of *cds1*^+^ (Kunoh et al. 2008). It is evident that the slow growth resulting from *pcf1*^+^ overexpression was partially suppressed by the H4.2 K8A K16G mutation (WT + *pcf1* vs. K8A K16G + *pcf1* in Figure 4C), when compared with growth of WT + Vec vs. WT + *pcf1* in Figure 4C. Results suggest that deacetylation of H4 by Clr6-HDAC can regulate function of Pcf1.

Deacetylation of H4 in HU-treated Δ*pcf1* mutant cells was comparable to that in the similarly treated wild-type cells (Figure 4D), suggesting that Clr6-HDAC activation could proceed adequately in the absence of Pcf1. This assumption led us to examine whether Clr6-HDAC activation was required for removal of Pcf1 from the chromatin. Figure 4E, showing that α-tubulin and histone H3 were detected only in the chromatin-unbound and -bound fractions, respectively, proved the proper performance of the assay. Recruitment of Pcf1-GFP to chromatin upon replication stress was abrogated by H4.2 K8A K16G mutation, which is clear in the comparison of the chromatin-bound fractions of wild type and H4.2 K8A K16G mutant cells at 4h in Figure 4E. Results led us to consider that removal of Pcf1 from chromatin in response to Clr6-HDAC-dependent H4-deacetylation might be sufficient to inactivate Cds1 upon replication stress. In another way, it is also considerable that Cds1 is recruited to remove Pcf1 from chromatin. Experiments are ongoing to test these possibilities and examine whether chromatin binds to Pcf1 when *clr6*^+^ is mutated and/or overexpressed.

## Discussion

Modification of chromatin structure caused by mutation of *pcf1*^*+*^ (Figure 1A) and enhanced sensitivity of Δ*pcf1* to HU (Figure 1C) suggest that Pcf1 could be required for replication-coupled nucleosome assembly in fission yeast. Genetic and biological analyses revealed that Pcf1 was crucial for sustained activity and chromatin recruitment of Cds1 upon replication stress (Figures 1D, 2, and 3A). In addition, overexpression of *pcf1*^+^ led to the recruitment of Cds1 to chromatin (Figure 3C and D) without the spontaneous phosphorylation of Cds1 (Figure 3B). Thus, we emphasize here that Pcf1 could be critical in the recruitment of Cds1 to chromatin and that phosphorylation of Cds1, which is regulated through the conventional checkpoint system, could not be required for this process, although it has been generally accepted that Cds1 phosphorylation is an essential key for activating the DNA replication checkpoint (Tanaka et al. 2001). Although Cds1 remains in an active state even after DNA replication is completed in the *clr6*-HDAC mutant (Kunoh et al. 2008), the Δ*pcf1* mutation directed the *clr6*-HDAC mutant cells toward mitosis (Figure 4B), suggesting that H4-acetylation-dependent maintenance of checkpoint could be regulated by Pcf1. Slow cell growth caused by overexpression of *pcf1*^+^ was suppressed by mutation of the H4 acetylation sites (Figure 4C). In addition, recruitment of Pcf1 to chromatin was lost with this mutation (Figure 4E). These results indicate the possibility that Pcf1 is regulated by H4 acetylation. Taken together all these data, it is inferred that deacetylation of H4 by Clr6-HDAC could regulate Cds1 via Pcf1. As mentioned, Haldar and Kamakaka (2008) reported that fission yeast cells deficient in Hst4-HDAC, which is responsible for H3-K56 deacetylation, were sensitive to DNA-damaging agents. Consistently, an effector kinase of DNA damage checkpoint, Chk1, was activated constitutively in the Hst4-HDAC-deficient cells (Haldar and Kamakaka 2008). These results and our current findings suggest that discontinuation of histone acetylation might generate signals to complete histone assembly on damaged or slowly replicating DNA so as to inactivate the S-phase checkpoint pathways.

RBAp48 that associates with the complexes including CAF-1, HDAC, and NURF in a broad range of species (Marheineke and Krude 1998; Nakayama et al. 2003) is thought to act as a liaison factor among these complexes. Therefore, RBAp48 could be one of the best candidates to regulate histone deposition and deacetylation, although it remains unclear whether RBAp48 is also required to maintain the DNA replication checkpoint. We conclude that nucleosome assembly by CAF-1, including Pcf1 and Clr6-HDAC, could be crucial for inactivating Cds1 after replication stress.

## Summary

In this study, we found that the deletion mutant of the *pcf1*^+^ gene, which encodes the large subunit of fission yeast CAF-1 failed to maintain the activity of Cds1, an effector kinase of DNA replication checkpoint. Consistently, chromatin recruitment of Cds1 in response to replication stress was also abolished by deletion of *pcf1*^+^. These results suggest the conserved function of CAF-1 could contribute to maintain checkpoint machineries in an active state. Intriguingly, Pcf1 overproduction enhanced the chromatin recruitment of Cds1 even in the absence of replication stress. In addition, the growth inhibition induced by the overproduction of Cds1 was significantly suppressed by the deletion of *pcf1*^+^, being consistent with the notion that CAF-1 might be crucial for sustained activation of the checkpoint effector Cds1. Importantly, chromatin recruitment of Pcf1 was negated by the mutation at the acetylation sites in H4 after replication stress. Furthermore, growth inhibition induced by Pcf1 overproduction was partially suppressed by H4 mutation. On the basis of these results, we propose that H4 deacetylation by Clr6-HDAC could modulate Cds1 activity by regulating recruitment of Pcf1 to chromatin.

## Materials and methods

### Strains, plasmids and media

The strains used in this study are the derivatives of *Schizosaccharomyces pombe h*^-^ 972 and *h*^+^ 975 (Beach et al. 1985). The mutant, *clr6-1*, and histone H4 mutant, H4.2 K8A K16G, were also used (Bjerling et al. 2002; Mellone et al. 2003). Standard fission yeast techniques and media were employed (Moreno et al. 1991). To construct pREP-*cds1*^+^ and pREP-*pcf1*^+^, the ORF of *cds1*^+^ and *pcf1*^+^ genes were independently amplified by PCR and cloned into pREP2 (provided by National BioResource Project, Japan). To repress transcription from the *nmt1* promoter of pREP2, the cells were grown in the EMM2 minimal medium supplemented with 4 μM thiamine. For examining hydroxyurea (HU) sensitivity of the cells, 12 mM HU was added to the medium. For spot assays, exponentially growing cultures were serially diluted five-fold, and each dilution was spotted onto agar plates containing HU and/or thiamine.

### Gene disruption and tagging

Gene disruption was performed as previously reported (Rothstein 1983). For myc8 or GFP tagging, an ~ 1-kbp fragment of the C-terminus region of *cds1*^+^ and *pcf1*^+^ genes were inserted into pYC11-myc8 and pYC11-6xGFP, respectively, and the resultant plasmids were introduced into both wild-type and mutant strains. Gene disruption and gene replacement were confirmed by southern blotting and immunoblotting.

### Cell extracts and immunoblot

For examining mobility of Cds1 and acetylated H4 on SDS-PAGE, exponentially growing cells of the wild-type and the Δ*pcf1* mutant were treated with 6 mM HU, then a cell extract was prepared from 0 to 6 h according to previous methods (Kunoh et al. 2008). For immunoblots, anti-c-myc (1:7500; Covance Inc., Princeton, NJ), anti-tetra acetylated histone H4 (1:5000; Bio-Rad AbD Serotec Ltd., Oxfordshire, UK) (described as tetra AcH4 in Figure 4D), anti-Tat1 (1:5000; generously provided by Dr. A. Baines), anti-GFP (1:5000; Roche Applied Science, Mannheim, Germany), anti-H3 (1:2500; Merk, Darmstadt, Germany) (described as tetra H3 in Figure 4E) antibodies served as the primary antibodies. Goat HRP conjugated-anti-mouse (1:25000; Life Technologies, Carlsbad, CA) and anti-rabbit antibody (1:25000; Life Technologies) were used as the secondary antibodies.

### Triton extraction and immunofluorescence microscopy

To monitor binding affinity of Cds1 to chromatin, Cds1-myc8 was extracted with TritonX-100 and immunostained as described previously (Zhao et al. 2003; Kunoh et al. 2008). Counting of cells with either segregated or Cds1-myc8-positive nuclei was replicated at three times (50 cells per replicate) in the field of view at x1000; mean values with SD are given in Figures 2D and 3D, respectively. The chromatin binding affinity of Cds1 was evaluated as the number of Cds1-myc8-positive nuclei per total number of DAPI-stained nuclei.

### MNase digestion of the bulk chromatin

The status of the global chromatins in Δ*pcf1* and *clr6-1* mutant was monitored using MNase (micrococcal nuclease) digestion assay. Isolation of bulk chromatin and MNase digestion were performed as described previously (Takahashi et al. 1992).

### Chromatin fractionation assay

To examine whether recruitment of Pcf1 to chromatin was affected by H4 acetylation, we first treated wild type and Δ*pcf1* mutant cells with 12 mM HU at designated times before chromatin fractionation. The fractionation procedure was generously provided by Drs. M. Sadaie and J. Nakayama (RIKEN, Kobe, Japan). Approximately 2.5 × 10^8^ cells of wild type or Δ*pcf1* mutant were harvested and washed in ice-cold STOP buffer (150 mM NaCl, 50 mM NaF, 10 mM ethylenediaminetetraacetic acid, and 1 mM NaN_3_). Cells were resuspended in 1 mL of PEMS buffer (100 mM PIPES, 1 mM EGTA, 1 mM MgCl_2_, 1.2 M sorbitol, pH6.9) containing 1 mg/mL each of lysing enzyme (Sigma, St. Louis, MO) and zymolyase 100 T (Seikagaku Co., Tokyo, Japan), followed by incubation at 37°C for 20 min. After washing out lysing enzyme and zymolyase 100 T with 1.2 M sorbitol, we then suspended the spheroplasts in HBS-T buffer (25 mM MOPS, 60 mM β-glycerophosphate, 15 mM MgCl_2_, 15 mM EGTA, 1 mM dithiothreitol, 0.2 mM Na_3_VO_4_, 1 mM phenylmethylsulfonyl fluoride, 1.2 M sorbitol, and 0.5% Triton X-100, pH7.2) and incubated on ice for 5 min. After centrifugation at 13,000 rpm for 20 min, the supernatant was saved as an unbound fraction. Then pellet was washed twice in HBS-T buffer, resuspended in the initial volume of HBS-T buffer, and saved as a chromatin fraction. Both fractions were subjected to immunoblots as described above. Proper performance of the assay was confirmed by using anti-α-tubulin and anti-H3 antibodies as loading controls. Fractionated Pcf1-GFP was detected by using the anti-GFP antibody.

### Septation index

In fission yeast, septum formation is one indicator of the completion of mitosis. Thus, we counted the number of cells with a septum in the field of view of a light microscope at x1000 to determine a septum index. The counts were replicated at least three times (50 cells per replicate).

## Abbreviations

CAF-1: Chromatin assembly factor-1
HDAC: Histone deacetylase
ATR: ATM and Rad3 related
Chk: Checkpoint kinase
Cds1: checking DNA synthesis
Rad: radiation sensitive
RBAp48: Rb-associated protein p48
NURF: Nucleosome Remodeling Factor
GFP: Green Fluorescent Protein
H3K56: Histone H3 Lysine 56
MNase: Micrococcal nuclease
HU: Hydroxyurea
WT: Wild type
Vec: Vector
Thi: Thiamine
DAPI: 4′,6-Diamidino-2-phenylindole
EGTA: 2-aminoethyl ether tetraacetic acid
H4.2 K8A K16G: Histone H4.2 Lysine 8 to Alanine and Lysine 16 to Glycine.
